# Supplementary material for: Adapterama III: Quadruple-indexed, double/triple-enzyme RADseq libraries (2RAD/3RAD)
Source: PeerJ. 2019 Oct 11;7:e7724. doi: 10.7717/peerj.7724 (PMC6791345; doi:10.7717/peerj.7724)
Supplement: File S4 — Document with instructions of how to reconstitute and anneal 2RAD/3RAD dried adapters to further use in 2RAD/3RAD libraries. [file peerj-07-7724-s004.docx]

How to Handle Plates with 3RAD v2 Adapter Aliquots

When you receive the adapters, there is 1.625 nmol of each oligo pair dried in each well, but they are **NOT** annealed. You will need to reconstitute them to the appropriate volume (64 µL -> 25 µM), anneal them, then dilute again (to 5 µM) & aliquot them.

Liquid for reconstitution & annealing (10 mM Tris pH 8, 0.1 mM EDTA, 100 mM NaCl):

For 50 mL of salty TLE, add the following to a 50mL conical:

40 mL dH_2_O

500 µL 1M Tris pH 7.5 to 8

20 µL 0.5M EDTA pH 8

1 mL of 5 M NaCl (or 5 mL of 1M NaCl)

Fill with distilled water to 50 mL mark.

**Protocol:**

1) Centrifuge the dry plates to get all the adapter to the bottom of the wells.

2) To limit contamination, peel back the foil cover from the plate one row at a time to reconstitute.

3) Add 64 µL of the liquid from above to each well.

-Use the pipet tip to help scrap the bottom of the well to dislodge any of the adapters that is stuck.

-Skloosh (pipette up & down) several times to mix.

-Wait a few minutes.

- Skloosh several more times to mix.

-Let the adapters sit in the liquid at room temperature for at least 5 minutes.

The adapters are now at 25 µM.

4) Anneal the adapters together:

-Use thermocycler to denature (95°C for 1 min.) & cool slowly (e.g., 0.1°C per sec.).

5) Dilute aliquots of the annealed adapters into new labeled strip tubes:

- Add 10 µL of annealed adapters to 40 µL of salty TLE (final conc. = 5 µM), for SIX separate strips. [Note: This will leave ~4 µL behind in the oligo plates (could be worth getting as much as possible for the Read1 Adapters – they are limiting; but the Read2’s are in excess, so leave that behind). Each strip (with 50 µL) can do FOUR full plates.]

6) Store the strip tubes of adapters at -20°C.

7) Before each use, take adapter aliquots out to thaw and **skloosh well before using!**
