# Supplementary material for: Adapterama III: Quadruple-indexed, double/triple-enzyme RADseq libraries (2RAD/3RAD)
Source: PeerJ. 2019 Oct 11;7:e7724. doi: 10.7717/peerj.7724 (PMC6791345; doi:10.7717/peerj.7724)

**Supplementary methods and results for 3RAD efficiency with low-quantity samples: a proof of concept**

We tested and compared our 3RAD protocol with the traditional ddRAD protocol (using the two flanking restriction enzymes from 3RAD and heat-killing after ligation) and 2RAD (using the two flanking restriction enzymes from 3RAD, but no heat-killing after ligation) with an array of sample dilutions.

To simplify the comparison between protocols, we used as template the pUC19 vector (NEB N3041S), which contains XbaI cut-site at position 423 and EcoRI cut-site at position 396. Our methods were as follows:

1. Amplified a ~500 bp fragment within the vector using the primers pUC19-215F-AAGGAGAAAATACCGCATCAGG and pUC19-774R-TAACCGTATTACCGCCTTTGAG and the following PCR recipe and conditions. We perform five PCR replicates.

5.0 μL 5x Kapa HiFi Buffer
1.0 μL dNTP’s (10 mM stock from Kapa kit)
34.5 μL dH_2_O (to make final total volume 50 μL)
2.0 μL Kapa HiFi DNA Polymerase (1 unit/μL from Kapa kit)
2.5 μL Forward Primer (5 μM)
2.5 μL Reverse Primer (5 μM)

5.0 μL pUC19 DNA 1 ng/μL

Cycling conditions: 95°C for 3 minutes (min), then 28 cycles of 95°C for 30 sec, 50°C for 30 sec, 72°C for 30 sec; and a final extension at 72°C for 5 minutes followed by holding at 4°C.

1. Pooled 5 PCR replicates and purified the PCR product with SpeedBeads with a 1:1 ratio and quantified with a Qubit fluorometer. This measured 24.8 ng/μL.
2. Made a 15 ng/μL aliquot.
3. Made a 10-fold dilution series from the 15 ng/μL aliquot. We used these five products (one stock and four dilutions) as input for the 3RAD, 2RAD and ddRAD libraries, plus a negative control which was the same TE1X used for the series of dilutions.
4. Digested with restriction enzymes using the following recipe:

|  | **3RAD** | **2RAD** | **ddRAD** |
| --- | --- | --- | --- |
| **CutSmart** | 1.5μL | 1.5 μL | 1.5 μL |
| **Adapters (2.5 μM**) | 2 μL/each | 2 μL/each | 2 μL/each |
| **DNA** | 3.3 μL | 3.3 μL | 3.3 μL |
| **Water** | 4.7 μL | 5.2 μL | 5.2 μL |
| **XbaI (20,000 U/mL)** | 0.5 μL | 0.5 μL | 0.5 μL |
| **EcoRI-HF (20,000 U/mL)** | 0.5 μL | 0.5 μL | 0.5 μL |
| **NheI (20,000 U/mL)** | 0.5 μL | 0 | 0 |

Cycling conditions: 37°C for 1 hour. For the ddRAD experiment, we heat-killed REs with a 20 min incubation at 65°C following digestion.

1. Immediately added the ligation mix (5 μL) to each sample. That mix was as follows:

   2.75 μL dH_2_O
   1.5 μL ATP (10 mM); note: rATP, NOT dATP
   0.5 μL 10x Ligase Buffer (ensure components are in solution [warm it up!])
   0.25 μL DNA ligase 400 units/μL

   Cycling conditions: 22 °C for 20 min., 37 °C for 10 min., 22 °C for 20 min., 37 °C for 10 min., 80 °C for 20 min., then hold at 10 °C.
2. Set up PCR reactions with the following recipe per-sample:

5.0 μL 5x Kapa HiFi Buffer
0.75 μL dNTP’s (10 mM stock from Kapa kit)
11.75 μL dH_2_O (to make final total volume 50 μL)
0.5 μL Kapa HiFi DNA Polymerase (1 unit/μL from Kapa kit)
2.5 μL i5 Primer (5 μM)
2.5 μL i7 Primer (5 μM)
10.0 μL input DNA from Step 6

Cycling conditions: 95 °C for 2 min, followed by 15 cycles of 98 °C for 20 sec, 60 °C for 30 sec and 72 °C for 30 sec; and a final extension at 72 °C for 5 min, holding after at 12 °C.

1. Ran the PCR products of each of the three experiments on a 2% agarose gel. For every DNA input concentration tested, the first well corresponds to 3RAD, the second to 2RAD, and the third to ddRAD library.


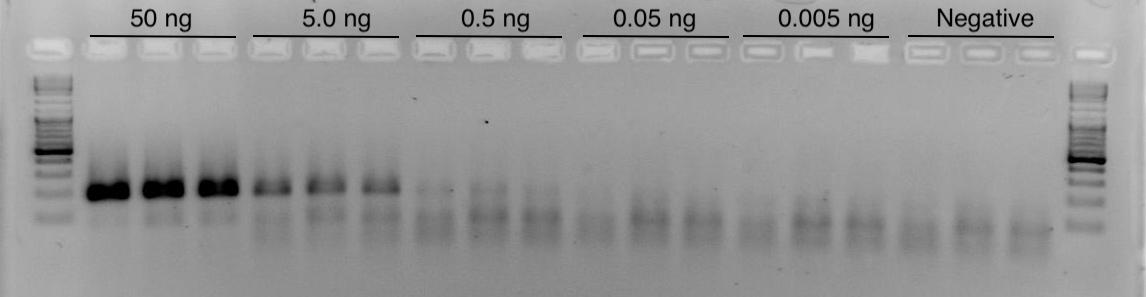

Supplement: File S5 — Step by step protocol with instructions on how the comparison of 3RAD, 2RAD and ddRAD methods was surveyed on pUC19 vector. [file peerj-07-7724-s005.docx]
