# Supplementary figures and images for: Adapterama III: Quadruple-indexed, double/triple-enzyme RADseq libraries (2RAD/3RAD)
Source: PeerJ. 2019 Oct 11;7:e7724. doi: 10.7717/peerj.7724 (PMC6791345; doi:10.7717/peerj.7724)

# 3RAD Quadruple-Indexed Libraries

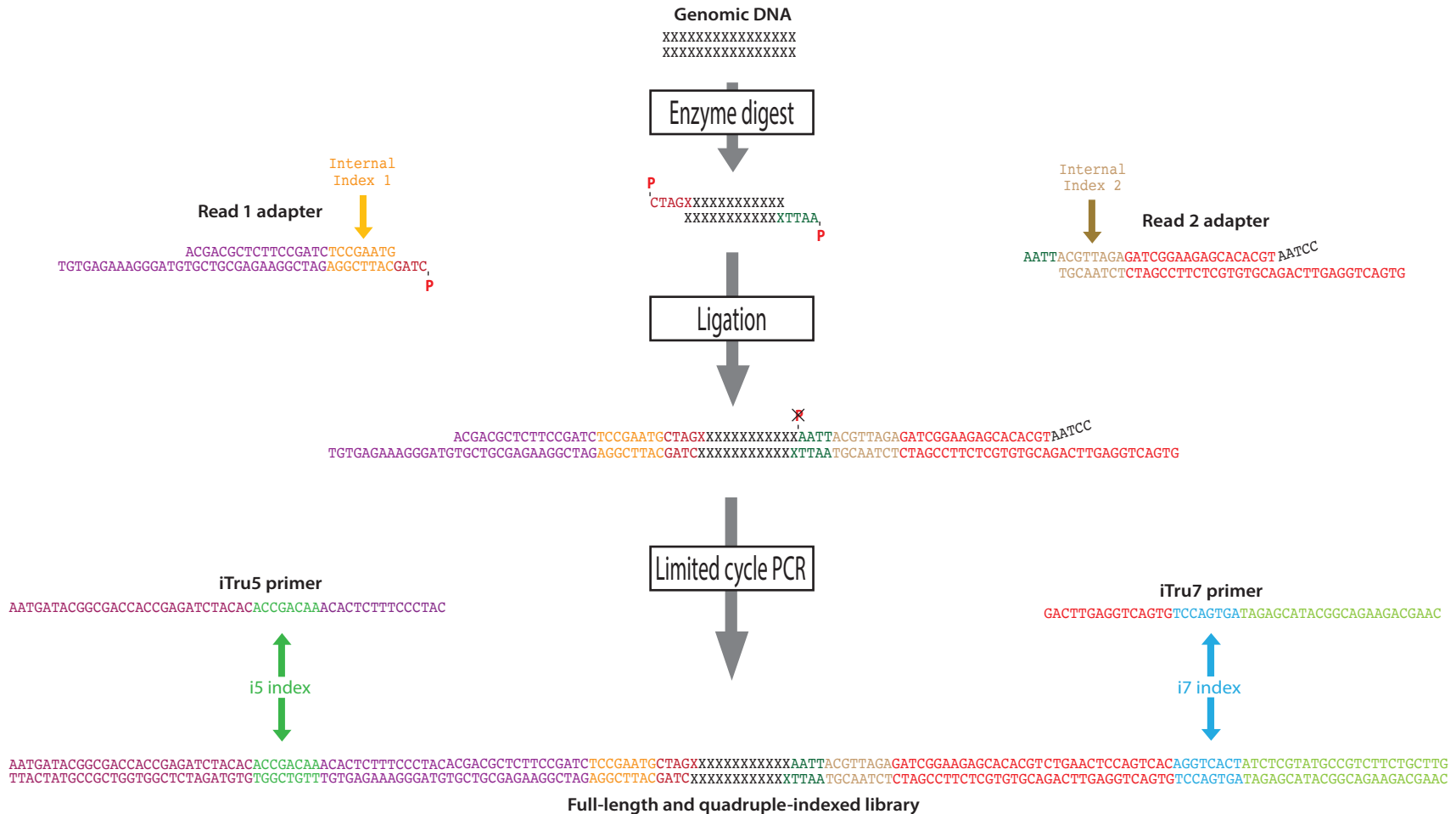

Supplement: Figure S1 — Detailed sequences for the workflow displayed in Fig. 1. 5′ phosphates are indicated with a red “P”. The Read 2 adapter lacks phosphates; thus, when it is ligated to the digested genomic DNA, a nick remains in the top strand (i.e., the phosphodiester bond between the genomic DNA and the adapter is missing, indicated by the “P” with an “X” through it). [file peerj-07-7724-s008.pdf]

# Quadruple-Indexed 3RAD Libraries

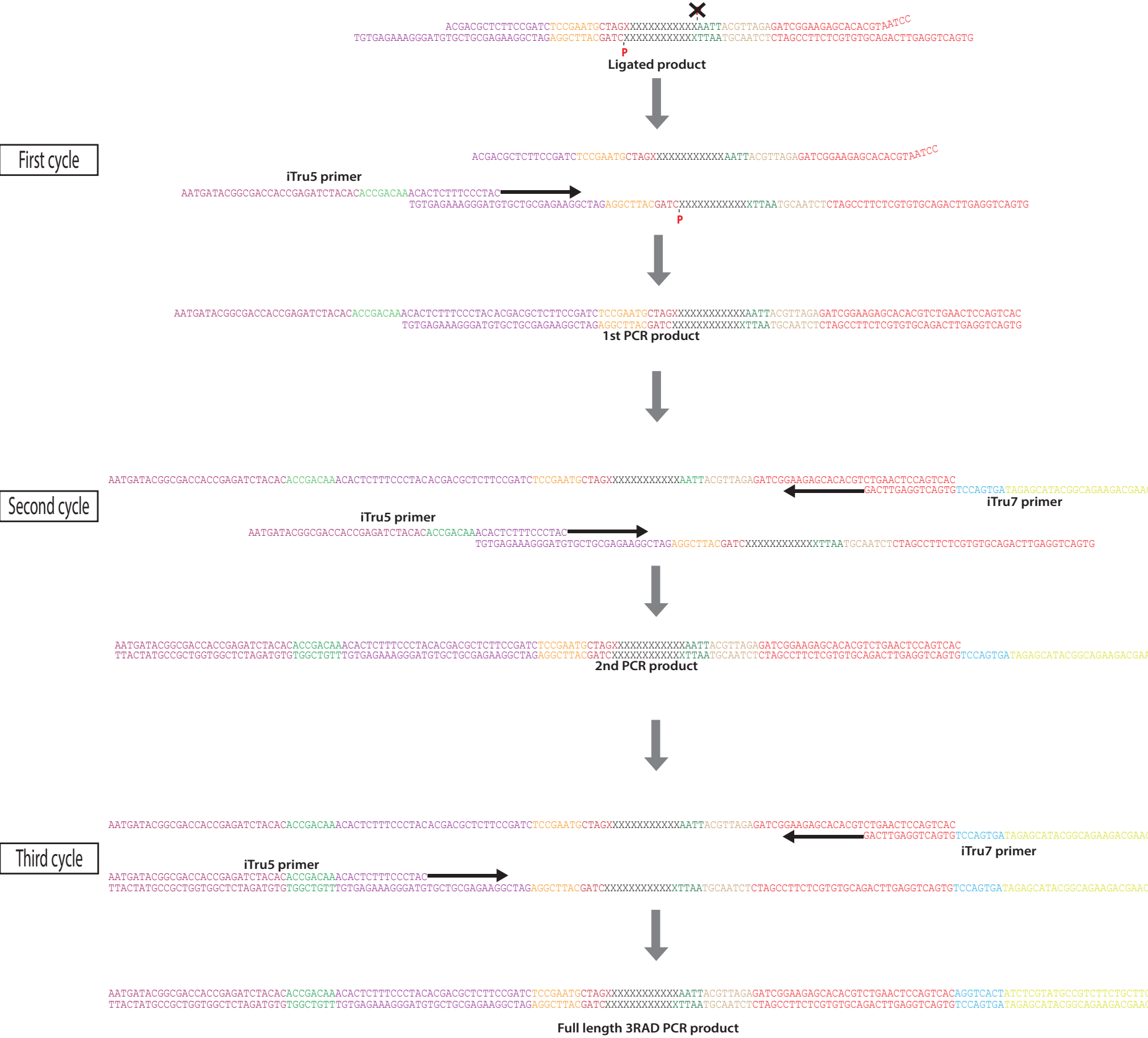

Supplement: Figure S2 — This figure demonstrates how only the bottom strand is used to form the fully-functional 2RAD/3RAD libraries. [file peerj-07-7724-s009.pdf]

Normalized Coverage (x)

Project

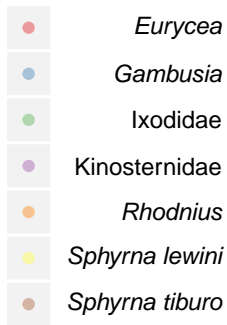

0

5

10

15

20

25

Genome Size (C-value)

60

40

20

Supplement: Figure S3 — We divided the average coverage for each sample by the total number of retained reads for that sample to obtain the normalized coverage. The Wisteria dataset is not included because the genome size is unknown. [file peerj-07-7724-s010.pdf]

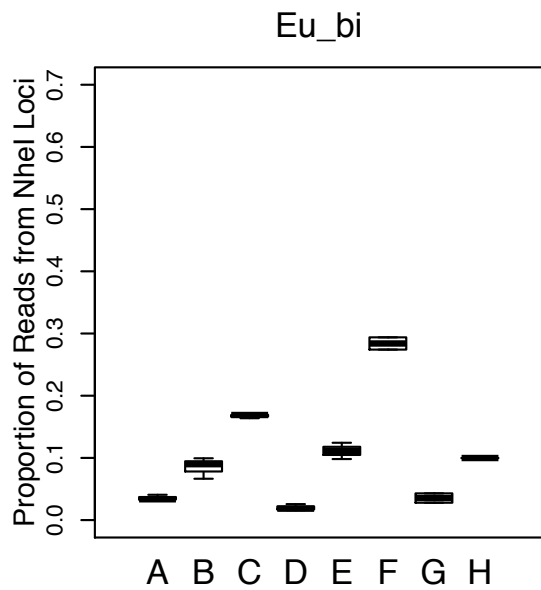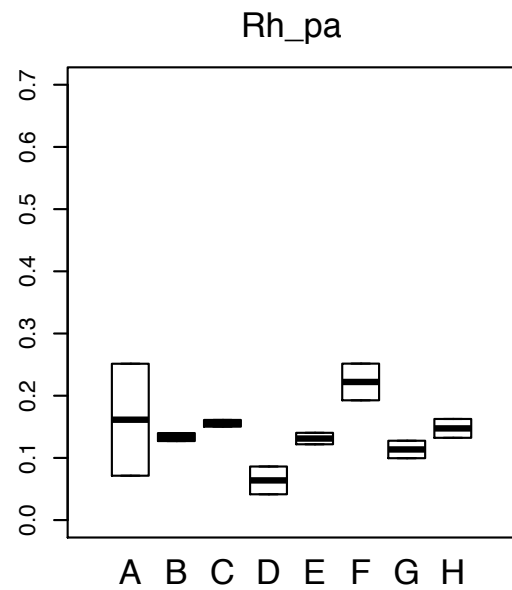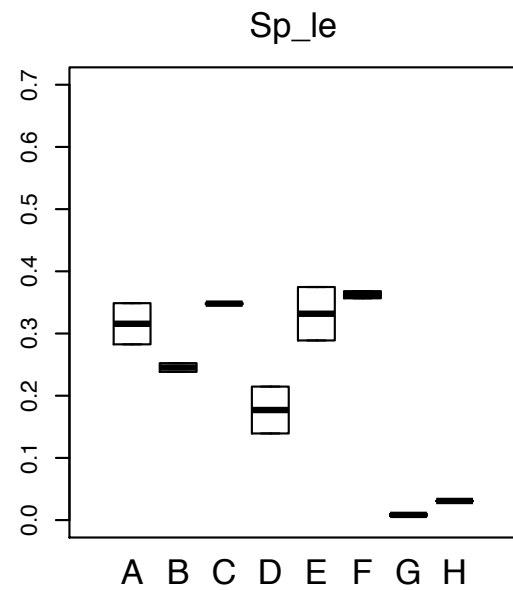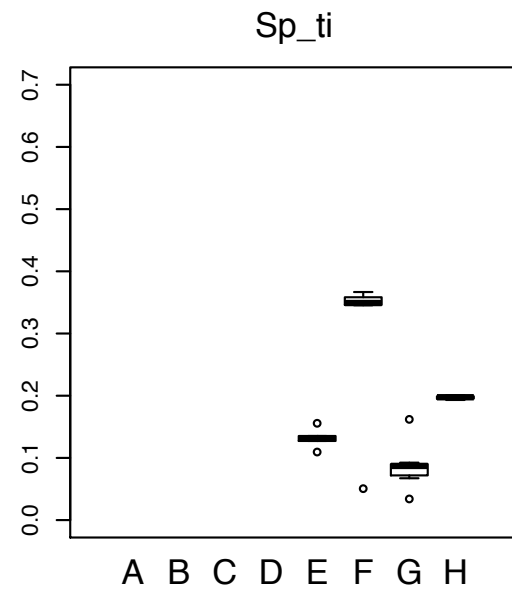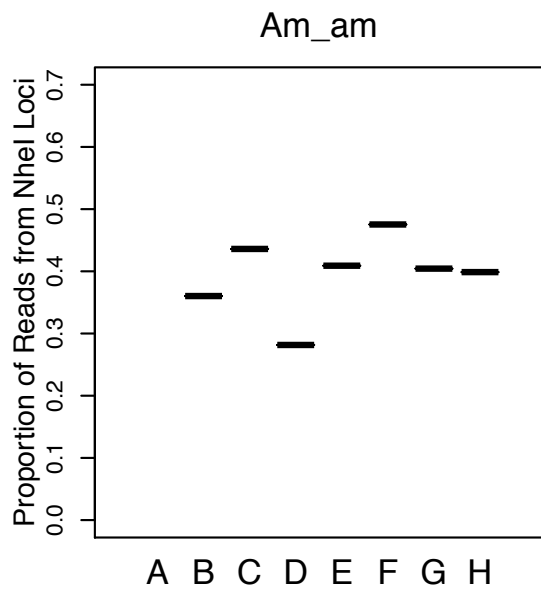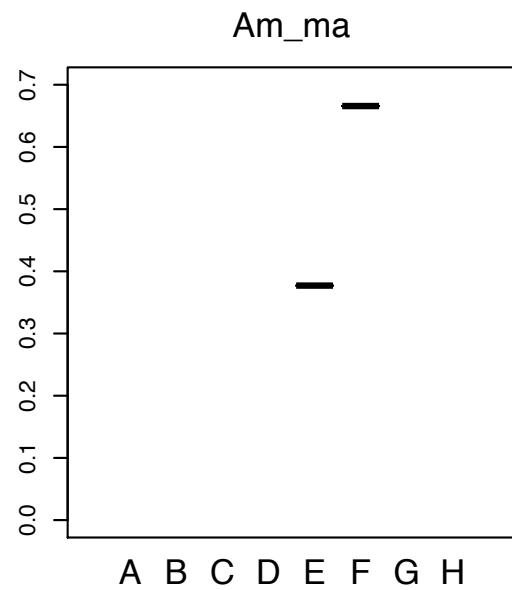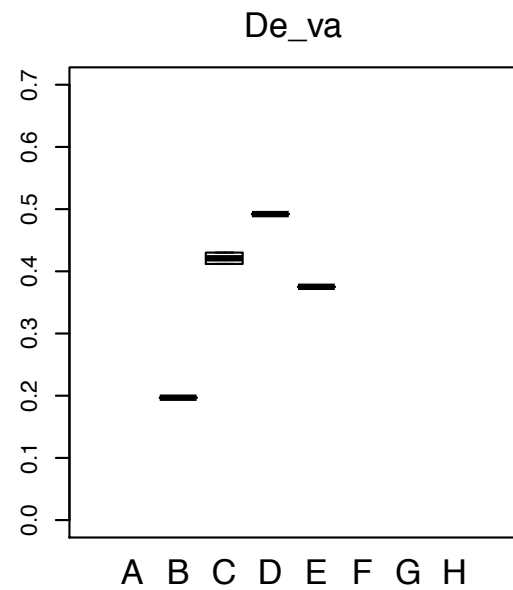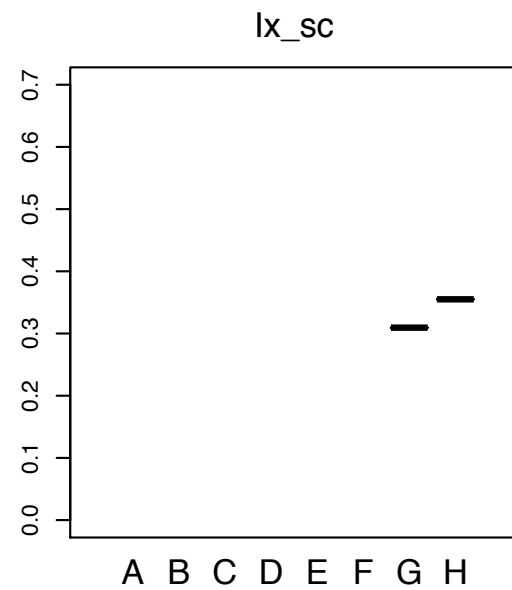

Adapter

Supplement: Figure S4 — The proportion of the total raw R1 reads derived from loci cut by NheI, rather than XbaI, in eight of our sample projects broken down by which adapter (i.e., NheI_A-H) was used. [file peerj-07-7724-s011.pdf]
